# Protection motivation theory and cigarette smoking among vocational high school students in China: a cusp catastrophe modeling analysis

**DOI:** 10.1186/s41256-016-0004-9

**Published:** 2016-06-15

**Authors:** Yunan Xu, Xinguang Chen

**Affiliations:** 1 University of Florida, Gainesville, Florida USA; 2 Wuhan University Global Health Institute, Wuhan, China; 3 Wuhan Center for Disease Prevention and Control, Wuhan, China

**Keywords:** Quantum change, Cusp catastrophe modeling, Cigarette smoking, Adolescents

## Abstract

**Background:**

Tobacco use is one of the greatest public health problems worldwide and the hazards of cigarette smoking to public health call for better recognition of cigarette smoking behaviors to guide evidence-based policy. Protection motivation theory (PMT) provides a conceptual framework to investigate tobacco use. Evidence from diverse sources implies that the dynamics of smoking behavior may be quantum in nature, consisting of an intuition and an analytical process, challenging the traditional linear continuous analytical approach. In this study, we used cusp catastrophe, a nonlinear analytical approach to test the dual-process hypothesis of cigarette smoking.

**Methods:**

Data were collected from a random sample of vocational high school students in China (*n* = 528). The multivariate stochastic cusp modeling was used and executed with the *Cusp Package* in R. The PMT-based Threat Appraisal and Coping Appraisal were used as the two control variables and the frequency of cigarette smoking (daily, weekly, occasional, and never) in the past month was used as the outcome variable.

**Results:**

Consistent with PMT, the Threat Appraisal (asymmetry, α_1_ = 0.1987, *p* < 0.001) and Coping Appraisal (bifurcation, β_2_ = 0.1760, *p* < 0.05) significantly predicted the smoking behavior after controlling for covariates. Furthermore, the cusp model performed better than the alternative linear and logistic regression models with regard to higher *R* ^*2*^ (0.82 for cusp, but 0.21 for linear and 0.25 for logistic) and smaller AIC and BIC.

**Conclusion:**

Study findings support the conclusion that cigarette smoking in adolescents is a quantum process and PMT is relevant to guide studies to understand smoking behavior for smoking prevention and cessation.

## Background

### Cigarette smoking in China

Cigarette smoking is recognized globally as one of the most preventable causes for morbidity and premature death. Tobacco killed about 6 million people a year and approximately 5 million of those deaths result from direct tobacco use. Furthermore, more than 600,000 innocent non-smokers died from exposure to second-hand smoke [46]. More seriously, the death resulting from smoking is expected to increase to more than 8 million a year by 2030 [16]. China is a biggest country of tobacco production and consumer in the world and the great hazard of cigarette smoking to public calls for better reorganization of cigarette smoking behavior and more effective prevention and intervention policies. Every year, there are approximately one million deaths attributed to tobacco use in China, and this number is estimated to reach 3 million by the middle of this century [19, 37]. In addition to the high rates of smoking among adults, more and more adolescents are now smoking [5, 23, 55]. Among the 104 million Chinese adolescents 12–17 years of age, 12.8 %–47.8 % have initiated smoking (33.3–47.8 % for males and 12.8–24.3 % for females) and 1.7–18.3 % are current smoking (15.0–18.30 % for males and 1.7–4.0 % for females) [6, 18, 47, 49].

Vocational high schools in China are designated to enroll students who fail to pass entrance examination for or elect not to advance to regular high schools because of concerns of poor academic performance from grade 1 to grade 9 as required by the Nine-Year Compulsory Education of China. Data from reported studies indicate that tobacco use among vocational high school students is more prevalent than students in regular high schools (26–30 % vs 8.6–14.6 % in past 30-day smoking) [1, 13, 48]. To reduce the high prevalence of tobacco use, more studies are needed to investigate the dynamic process of and factors associated with the increased risk of tobacco use. However,results from a review of the literature in both Chinese and English indicate that few tobacco-related studies have focused on vocational high school students.

### Protection motivation theory and cigarette smoking

Protection Motivation Theory (PMT) provides a cognitive conceptual framework to investigate tobacco use among adolescents. The cognitive process plays a key role in the process of decision-making, leading to behavioral change. PMT consists of two pathways: Threat Appraisal and Coping Appraisal, and it is the interaction of the two that determines the likelihood whether an adolescent will smoke or not. Threat Appraisal serves as an evaluation of maladaptive behaviors, and it includes four constructs in two groups: Perceived Threat (severity and vulnerability) and Perceived Rewards (intrinsic rewards and extrinsic rewards). Coping Appraisal serves as an evaluation of a person’s ability to manage and avoid the threat, and it includes three constructs also in two groups: perceived efficacy (response efficacy and self-efficacy) and perceived cost (response cost). Those who perceive greater threat from smoking and have higher coping ability to adapt non-smoking behaviors are less likely to smoke. Some studies have reported the successful utilization of PMT in predicting cigarette smoking among adolescents in China [45, 54]. In a study, a measurement scale for assessing PMT was reported with adequate reliability and validity [32]. Therefore, the Threat Appraisal and Coping Appraisal, derived from the Protection Motivation Theory, were used to predict smoking behavior in this study.

### Quantum nature of health behavior

The term quantum behavior change (QBC) is derived from quantum theory in physics (e.g., dual-characteristics of wave and particle of a photon) to describe the dual characteristic of human behavior changes – a linear and continuous process and a nonlinear and discrete process [26, 36, 38]. For example, in an investigation of 918 participants who reported having made at least one quit attempt in the United Kingdom, researchers found that 48.6 % of these participants’ quitting behavior did not occur through a planning and reasoning process [51]. A number of studies have indicated that the process for an adolescent to engage in substance use may be more discrete and sudden than continuous, gradual and analytical, including use of tobacco [4, 31, 35, 51], alcohol [10, 20, 25, 33], and other drugs [20].

However, the past research and prevention programs were conducted mainly based on the implicit or explicit assumption that human behaviors were logical, analytical and premeditated, a process of continuous behavior change (CBC). Only considering CBC element of behaviors greatly limits our correct understanding of health and risk behaviors and thus compromises the effects of prevention activities. Results from a previous study based on CBC indicated that PMT only explained 27 % variance of a smoking behavior measure [54]. PMT is a well-established cognitive theory and it has been used to predict a number of health-related behaviors in research [52, 54]. The relatively small variances explained by PMT through a CBC-based multiple linear regression method suggest the need for new analytical methods to assess the role of PMT in explaining human behaviors. Given the strengths of PMT in guiding behavioral research, we believe that the relatively small amount of variances explained by PMT in the reported studies is primarily due to the application of the CBC-based linear analytical approach. To test this hypothesis, in this study we modeled adolescent smoking behavior using a QBC-based method-cusp catastrophe modeling, assuming that change in adolescent smoking behavior is a quantum process.

### Dual process theory of cognition

**Fig. 1:**
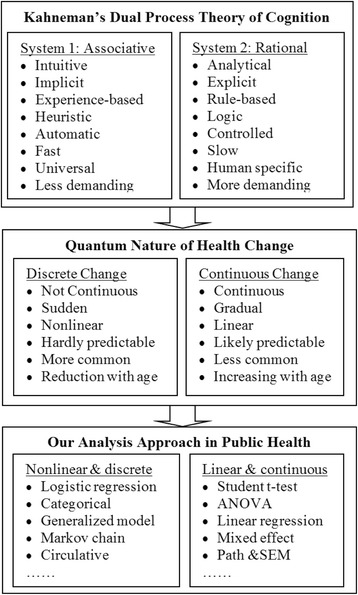
Two systems of the dual process theory. Behaviors are governed by two systems of cognitions, a nonlinear and discrete process and a linear and continuous process. System 1 works quick, for repeated daily events and emergency, less cognitive demanding while system 2 performs more slowly with a sequential thinking guided by logic and evidence

The quantum nature of health risk behavior can be explained by the Dual Process Theory proposed by Kahneman [28], the winner of the 2002 Nobel Memorial Prize in Economic Sciences. Based on psychological research regarding associative thinking [14, 27, 41], Kahneman posited a dual process theory consisting of two related systems (broadly termed as System 1 and System 2) to explain people’s behavioral decision and choices [28]. Figure 1 shows the two systems of dual process theory.

*System 1*, also known as intuitive, implicit, experiential, associative or heuristic system, consists of a set of “automatically operated” subsystems to solve problems by relying on prior knowledge, experiences and belief. Therefore, System 1 works quick, for repeated daily events and emergency, less cognitive demanding, can be completed under stressful and emotional conditions, and with more focus on the immediate rewards. On the contrary, *System 2*, also known as rational, explicit, rule-based, self-controlled, logic or analytical system, performs more slowly with a sequential thinking guided by logic and evidence. Therefore, System 2 has a higher cognitive demanding than System 1, and cannot perform well under the influence of stressful and emotional conditions [28, 42].

### Dual process theory links PMT with smoking behavior

The Dual Process Theory of cognition has been used in research to successfully explain “failure in rational thinking” in behavioral economics [14, 15, 28, 41] and reported life satisfaction [29, 40]. This theory may also be valid to explain cigarette smoking behaviors. For example, although about 80 % of smokers are aware that smoking is not good, many of them continue to smoke; many people smoke not because they are unaware of the harms from smoking [53]. The failure of rational thinking in explaining smoking behavior among some people could be due to certain dysfunction of the analytical System 2 with the intuitive System 1 dominating the behavior process.

As a cognitive process for decision-making, the relationship between PMT and smoking behavior may also consist of a dual process. The Threat appraisal process of PMT is more knowledge-based than intuitive and therefore it will be more likely to be operated through System 2 that is analytical. The Coping appraisal of PMT, on the other hand, is more belief-based than analytical, therefore it will be more likely to be operated by System 1 that is intuitive. Adolescence as a critical period in life is characterized by rapid and unevenly paced development, and their behaviors are often more intuitive and impulsive (System 1) than rationale and analytical (System 2) [3, 12]. For students with a balanced function of System 1 and System 2, they will be able to evaluate and analyze the threat from smoking and their ability to deal with the consequences from smoking; the relationship between PMT constructs and smoking measures would be continuous. However, for adolescents whose System 1 dominates System 2, they may not be able to assess the threat of tobacco and their ability to cope; the observed smoking behavior of these adolescents would be discrete and sudden, and not in consistent with the rational process of thinking. Up to date, no reported studies ever used this potential dual cognitive process of PMT to predict tobacco use behavior in adolescents.

### Quantify dual-process with cusp catastrophe model

Few statistical methods available so far can simultaneously characterize a discrete and a continuous process in one model but cusp catastrophe model provides an option. The cusp catastrophe model was first proposed by Tom [44] and has been adopted for use to in economics [39] and health behaviors [8, 20, 21]. The central idea of a cusp catastrophe is that status *z* of a behavior is determined by a potential function *V*(*z*; *x*, *y*) with two control variables *x* (asymmetry) and *y* (bifurcation):

1 $$ V\left(z;x,y\right)=\frac{1}{4}{z}^4+\frac{1}{2}y{z}^2+xz $$

With an equilibrium plane as the first derivative that is set to zero:

2 $$ \frac{dV\left(z;x,y\right)}{dz}=-{z}^3+yz+x=0 $$

The deterministic cusp model was extended to a stochastic process by adding a Weiner process for likelihood estimation [17]. In the extended stochastic cusp modeling approach, the control variables *x* and *y* and dependent variable z can each be a single observed variable (such as age, days smoked, number of cigarettes smoked per day), or a latent factor consisting of i observed variable *x*, j observed *y* and *k* observed z through a linear combination:

3 $$ x={\alpha}_0+{\alpha}_1{x}_1+{\alpha}_2{x}_2+\dots +{\alpha}_i{x}_i $$

4 $$ y={\beta}_0+{\beta}_1{y}_1+{\beta}_2{y}_2+\dots +{\beta}_j{y}_j $$

5 $$ z = {w}_0 + {w}_1{z}_1+{w}_2{z}_{2+}\dots +{w}_k{z}_k $$

**Fig. 2:**
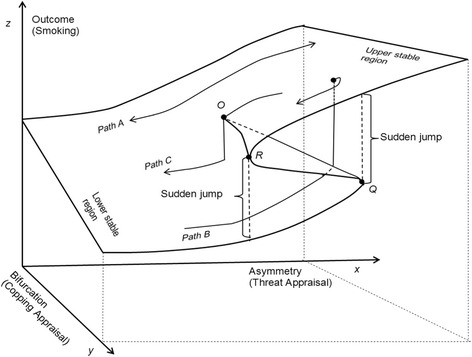
Proposed Cusp Model for Adolescent Cigarette Smoking. This figure presents the dynamic change among three variables along with the equilibrium plane of a cusp model. The argument x as asymmetry or normal control factor decides that the z changes asymmetrically from one mode to the other mode with x increases. The argument y as the bifurcation or splitting control factor causes the z to split and bifurcate from smooth changes to sudden jumps with y increases. When y is in any situation where y < O, there is a continuous and approximately linear relation between the asymmetry variable x and outcome z (see path A in Fig. 2). However, when the bifurcation variable y is sufficiently large to pass O, change in outcome z will be no longer continuous. Path B shows that when x increases to pass an ascending threshold line O-Q, z will suddenly leap from the lower stable region to upper stable region; Path C shows a sudden drop in outcome z as x decreases to reach and pass the descending threshold line O-R. In this study, x = Threat Appraisal, y = Copping Appraisal, and z = Cigarette Smoking

The cusp modeling can simultaneously characterize a discrete and a continuous process (see Fig. 2). The argument *x* as asymmetry or normal control factor determines change in *z* to be asymmetrical from one mode to another with *x* increases. The argument *y* as the bifurcation or splitting control factor causes the *z* to split and bifurcate from smooth change to sudden jump at certain levels of *x* as *y* increases. When *y* is in all locations where *y* < O, there is a continuous and approximately linear relation between the asymmetry variable *x* and the outcome *z* (Path A in Fig. 2). However, when the bifurcation variable *y* is sufficiently large to pass O, change in the outcome *z* will no longer be continuous. Path B shows that when x increases to pass an ascending threshold line O-Q, *z* will suddenly leap from the lower stable region to upper stable region; while Path C shows a sudden drop in *z* as *x* decreases to reach and pass the descending threshold line O-R. The utilization of the cusp modeling will assist us to better describe and quantify the data with a discrete and a continuous process while evidence obtained from such analysis, in turn, can be used to advance theories and models to better explain a behavior with quantum nature.

### PMT-based cusp model of cigarette smoking

The purpose of this study is to test the proposed cusp model in describing quantum process of cigarette smoking among Chinese adolescents guided by PMT. The ultimate goal is to provide new data and analytical approach to advance tobacco research and support more effective tobacco use prevention.

To identify cigarette smoking follows a quantum process, a cusp catastrophe model based on PMT were constructed. Although we hypothesize the PMT functions in adolescent smoking behavior through a discrete and a continuous cognitive process, there is a lack of existing evident to determine which of the threat appraisal and coping appraisal should be classified, for purposes of the modeling, as an asymmetry or bifurcation. However, findings from previous research suggested that when all the predictor variables as both symmetry and bifurcation were put into the modeling analysis, the predictor variable associated with the asymmetry variable was statistically significant only if it was modeled as asymmetry; this same effect applied to the bifurcation variable [17]. Therefore, in the modeling analysis, both the threat appraisal and coping appraisal were simultaneously put into the linear combination of asymmetry and bifurcation to test and describe their respective functions in deciding smoking behavior.

The asymmetry control factor x as a linear combination of the predictor variables of threat appraisal and copping appraisal were initially defined as:

6 $$ x={\alpha}_0+{\alpha}_1\left(\mathrm{threat}\ \mathrm{appraisal}\right) + {\alpha}_2\left(\mathrm{copping}\ \mathrm{appraisal}\right) $$

Age and gender should be included as covariates because of significant differences in cigarette smoking by gender and age among adolescents in China [7, 50]. However, because of narrow range of ages among the participants (15–18 years old), cigarette smoking may have higher homogeneity in the same grade and higher heterogeneity in the different grade compared to different ages in the study. Research has been confirmed as significant association between school grades and smoking behaviors [24]. Therefore, gender and grade as covariates are included into the equation (6):

7 $$ x={\alpha}_0+{\alpha}_1\left(\mathrm{threat}\ \mathrm{appraisal}\right) + {\alpha}_2\left(\mathrm{copping}\ \mathrm{appraisal}\right) + {\alpha}_3\left(\mathrm{gender}\right) + {\alpha}_4\left(\mathrm{grade}\right) $$

Where the intercept coefficient *α*_0_ assesses the mean effect of all predictor variables on the dependent variable of cigarettes smoking (z); *α*_1_ through *α*_4_ assess the independent effect of the variables on the dependent variable of cigarettes smoking (z).

The bifurcation factor y as a linear combination of the predictor variables of threat appraisal and copping appraisal are defined as:

8 $$ y={\beta}_0+{\beta}_1\left(\mathrm{threat}\ \mathrm{appraisal}\right) + {\beta}_2\left(\mathrm{copping}\ \mathrm{appraisal}\right) $$

Where the intercept coefficient *β*_0_ represents the mean on the dependent variable of cigarettes smoking (*z*); *β*_1_ and*β*_2_ assess the independent effect of the two variables on the dependent variable of cigarettes smoking (*z*).

The dependent variable of cigarettes smoking (z) is also defined as a linear equation:

9 $$ z = {w}_0 + {w}_1\left(\mathrm{cigarettes}\ \mathrm{smoking}\right) $$

Where *w*_0_ and *w*_1_ represent the mean level and change in cigarettes smoking along with the two latent control variables *x* and *y* defined above.

## Methods

### Participates

The study was conducted among vocational high school students in Wuhan, China. Vocational high schools in China provide three-year education focusing on employment-oriented trainings. Students in grades 1 and 2 were included, and students in grade 3 were excluded because they were preparing for graduation. After considering school size, diversity of training programs and other key factors, we chose a school with medium size and multiple occupation directions. Among the total 35 grade-1 and grade-2 classes, 17 were randomly selected using the random digit method. All students (*n* = 556) in the selected classes were invited to participate, 553 (99.5 %) provided parental consent and student asset were entered into final study and 3 refused to participate were excluded.

### Data collection

Participants completed the questionnaire survey in about 25–35 min. The survey was administered in classroom setting by trained data collector from Wuhan Center for Disease Control and Prevention (Wuhan CDC). The research protocol was approved by Human Investigation Committee at Wayne State University, the United States, and the Institutional Review Board Wuhan CDC.

Outcome variable used for this study was the frequency of cigarette smoking in the past month. This variable was assessed based survey response from the participants to the question: “Please think about the past 30 days (one month), including today, have you smoked any cigarettes during this period?” Response options were 1 = every day or almost every day, 2 = weekly but not every day, 3 = occasionally, and 4 = never.

The Threat Appraisal and the Coping Appraisal were assessed using the PMT Scale developed and validated for adolescents in China [32]. The PMT Scale consists of 7 constructs and 21 items, with 3 items per construct. A 7-point Likert-type response was used for item assessment with 1 = definitely disagree, and 7 = definitely agree (Table 1). Cronbach’s α =0 .75 for Threat Appraisal and 0.71 for Coping Appraisal. Difference between perceived threat and perceived rewards was calculated to assess Threat Appraisal and the higher scores indicated a perception of greater threat from smoking. Likewise, difference between perceived efficacy and perceived cost was calculated to assess Coping Appraisal and the higher scores indicated greater coping ability to adapt non-smoking behaviors.

**Table 1:** Promotive motivation theory scale for adolescent smoking

Threat appraisal	
Perceived Threat	Severity
	1 The earlier a person starts smoking, the greater the harm
	2 More smokers get sickness than nonsmokers
	3 Smokers died earlier than nonsmokers
	Vulnerability
	4 I would become addicted if I smoke
	5 I would get sick if I smoke
	6 If I smoke, I may die earlier
Perceived Rewards	Extrinsic Rewards
	7 Smokers look cool and fashionable
	8 Smoking is good for social networking
	9 The life of a smoker is happier than a nonsmoker
	Intrinsic Rewards
	10 Smoking makes people feel comfortable
	11 Smoking helps people concentrate
	12 Smoking enhances brainwork
Copping appraisal	
Perceived Efficacy	Response Efficacy
	13 People will feel good by not smoking
	14 People will be less likely to get disease if they do not smoke
	15 Quitting smoking is good for disease recovery
	Self-Efficacy
	16 No one could persuade me if I do not want to smoke
	17 Even if all who are around me smoke, that does not mean I must smoke
	18 I can refuse even if a relative or friend asks me to smoke
Perceived Cost	Response Cost
	19 A person may be isolated if he or she does not smoke
	20 Refusing a cigarette offer is very impolite
	21 One will miss the enjoyment if he or she does not smoke

### Data analysis

Survey data were entered into computer in double-entry way to maximize accuracy of data. Data processing and statistical analysis were completed using the R 3.2.1 and *P* < 0.05 indicated there was statistical significance. We completed the analysis of cusp catastrophe model by using the R packages “Cusp” which is a free and open computing program. Data-model fitting was evaluated using Akaike’s Information Criterion (AIC), Bayesian Information Criterion (BIC) and R^2^. Models with smaller AIC, smaller BIC, and higher R^2^ indicates a better fit in describing the cigarettes smoking.

## Results

### Sample characteristic

A total of 553 students participated in the study and 528 were included in the final analysis. Twenty five students who did not provide effective questionnaires were excluded. Among these students, 268 were girls (51 % of the sample) and 260 were boys. Two hundred and ninety students (55 %) were from grade 1 and 238(45 %) were from grade 2 aged 15–18 years old (M = 16.27, SD = 0.98). Most students’ fathers (*n* = 402, 76 %) and mothers (*n* = 358, 68 %) attained middle school/technical secondary school or higher education level. Approximately three quarters of the participants (74 %) reported no smoking in the past month and 63 (12 %) reported occasional smoking. Students who reported weekly smoking were almost equal to those who smoked every day or almost every day, with 38 (7 %) and 34(7 %), respectively. Of the male participants, 118 (45 %) smoked on average one or more cigarettes per day in a typical day during the past month and 33 (13 %) smoked every day; of the female participants, 17 (6 %) smoked cigarettes in the past month and none of them reported daily smoking.

### Cusp modeling analysis of cigarettes smoking

Table 2 shows the comparison between multiple linear regression, the nonlinear logistic model and cusp catastrophe model. The cusp catastrophe model was comparatively superior to the linear model and the nonlinear logistic model with higher R^2^ (0.82) and lower AIC (622) and BIC (665) for cusp models than for the alternative linear (*R* ^*2*^ = 0.21, AIC = 1233, BIC = 1258) and logistic models (*R* ^*2*^ = 0.25, AIC = 1208, BIC = 1246).

**Table 2:** Cusp catastrophe modeling of reported days of smoking during the 30-day period prior to the survey, including the survey day

Model variables	Asymmetry	Bifurcation	Days smoked
Estimated parameter			
Intercept	*α*_*0*_ = −1.4314**	*β*_*0*_ = 2.9907**	*w*_*0*_ = −3.4534**
Threat appraisal	*α*_*1*_ = 0.2168**	*β*_*1*_ = −0.0010	*w*_*1*_ = 1.4228**
Coping appraisal	*α*_*2*_ = −0.0010	*β*_*2*_ = 0.1517	
Model fit	Cusp	Logistic	Linear
* AIC*	736	1318	1332
* BIC*	770	1348	1349
* R* ^*2*^	0.81	0.07	0.04
Estimated parameter			
Intercept	*α*_*0*_ = 0.3515	*β*_*0*_ = 2.7717**	*w*_*0*_ = −3.4748**
Threat appraisal	*α*_*1*_ = 0.1987**	*β*_*1*_ = −0.0288	*w*_*1*_ = 1.4217**
Coping appraisal	*α*_*2*_ = −0.0466	*β*_*2*_ = 0.1760*	
Gender (if female)	*a*_*3*_ = 1.3050**		
Grade	*a*_*4*_ = 0.0120		
Model fit	Cusp	Logistic	Linear
* AIC*	622	1208	1233
* BIC*	665	1246	1258
* R* ^*2*^	0.82	0.25	0.21

Note:

*p < 0.05

**p < 0.001

Before adjusted by gender and grade, threat appraisal was significant only as asymmetry control factor (*α*_*1*_ = 0.2168, *p* < .001); although coping appraisal did not display statistical significant as both asymmetry and bifurcation factor, it had greater influence on cigarettes smoking as bifurcation variable (*β*_*1*_ = −0.0010, *β*_*2*_ = 0.1517). After adjusted by gender and grade in equation (7), threat appraisal and coping appraisal were significant only as asymmetry (*α*_1_ = 0.1987, *p* < .001) and bifurcation factor (*β*_*2*_ = 0.1760, *p* < .05), respectively.

Similar to the logistic regression and linear regression model, a positive value of coefficient from the cusp modeling indicates an increased risk to smoke cigarettes as the predictor variable increases. In this study, both threat appraisal and coping appraisal were positively associated cigarette smoking (*α*_1_ > 0 and *β*_*2*_ > 0). A reverse coding was sue such that a higher value indicating less smoking (e.g., 1 = daily smoking and 4 = never). Therefore, the coefficients could be interpreted that the threat appraisal and coping appraisal were positively associated with reduced risk of cigarette smoking. From the results, the risk of cigarette smoking was lower for adolescents who perceived greater threat from smoking (higher threat appraisal) and higher for adolescents who perceived less threat (lower threat appraisal). When coping appraisal (bifurcation) is at relatively lower levels, the relationship between threat appraisal and risk of cigarette smoking was gradual and continuous (see Path A in Fig. 2). When coping appraisal was high, the association between threat appraisal and cigarette smoking became discontinuous and complicated: (a) coping appraisal may interact with higher threat appraisal to maintain low likelihood of cigarette smoking (upper stable region in Fig. 2); (b) coping appraisal may become dysfunctional with lower scores of threat appraisal, resulting in sustained high risk of cigarette smoking (lower stable region in Fig. 2); (c) as threat appraisal varied at descending threshold or ascending threshold, sudden drop or sudden increase in the risk of smoking appeared in response to even small and subtle changes in either threat appraisal and coping appraisal (see Path B and C in Fig. 2).

## Discussion

In this study, we reported our research results regarding the relationship between the two pathways of PMT and cigarette smoking. The advanced stochastic cusp catastrophe modeling was used to characterize the quantum nature of cigarette smoking behavior with data collected from a random sample of vocational high school students. Findings of the research indicate that the change in cigarette smoking among adolescent is a quantum process that contains a *continuous and linear process* and a *discrete and nonlinear process*. The Threat Appraisal as an asymmetry variable and Coping Appraisal as a bifurcation variable can successfully predict the risk of cigarette smoking among Chinese adolescents in vocational high schools. When coping appraisal level is low, the impact of the threat appraisal on the risk of cigarette smoking is continuous, gradual, and linear. As the level of coping appraisal increases, the relationship between the threat appraisal and risk of cigarette smoking becomes discrete, sudden and nonlinear. Additionally, the AMT consisting of threat appraisal and coping appraisal can significantly better predict cigarette smoking if analyzed using the cusp models compared to the linear and logistic regression models. The lower AIC and BIC and higher R^2^ from the cusp model than from the corresponding alternative linear models reveal the superiority of cusp catastrophe modeling than linear and logistic model to characterize cigarette smoking behavior as quantum change.

Findings from the study reinforce the importance to incorporate the quantum behavior change (QBC) paradigm to advance the continuous behavior change (CBC) paradigm in investigating tobacco use among adolescents. Despite the obvious significance of PMT in understanding health behavior, including tobacco use, previous research tends to overlook the role of quantum process [45, 54]. In this research, we have demonstrated that the linear and logistic regression models explain respectively 21 % and 25 % of the variance of smoking behavior, significantly lower than 82 %, the variances explained by the cusp model. This finding suggests it is not the theory, but the QBC-based analytical method that help realize the power of PMT in predicting adolescent smoking. The QBC-based methods make a distinction between the two PMT pathways of Threat Appraisal and Coping Appraisal, further enhancing our understanding of PMT at the cognition level. The effect of the threat appraisal pathway as an asymmetry factor on likelihood of cigarette smoking is gradual, continuous, and linear. This suggests that the threat appraisal pathway is likely to be operated through System 2, because System 2 is in charge of decisions that are continuous and logic [28]. However, as the level of coping appraisal increases, the linear and continuous effect of the threat appraisal pathway can be interrupted and switch to sudden, discontinuous and discrete mode. Coping appraisal leads to discrete and discontinuous change, it can therefore be considered to be operated by System 1 that is discrete and intuitive [42].

The important findings facilitate effective implement of evidence-based policies and prevention activities. For adolescents, a long-term and gradual process through System 2 is needed to accumulate knowledge on the harm of health risk behavior, including tobacco use. Therefore changes in knowledge and their impact on behavior should be gradual and continuous, and improvement in tobacco-related knowledge is necessary for smoking prevention and quitting. However, when in situations an adolescent has to cope with pressure for smoking (e.g., social norm, peer pressure, and routine habits), the automatic and intuitive process of System 1 may dominate the analytical and rational process of System 2, resulting in discrete and sudden behavior change (unplanned quit or continue smoking despite knowing the harm of smoking). It is clear that it is the interaction between the two pathways of PMT that underpins the process of smoking behavior among adolescents. For adolescents with high levels of coping ability, increasing their capability to assess the risk of smoking should be considered as priority for smoking prevention and quitting; whereas for adolescents with low levels of coping, it is of the equal significance to strengthen their ability to assess the risk of smoking and cope with smoking prevention and quitting.

Findings from this research expand application of cusp catastrophe modeling and add evidence supporting utilization of cusp modeling to characterize health behaviors with QBC nature. The cusp catastrophe modeling has been successfully applied in various fields to explain the process of quantum change,such as physical, biological, social, psychological and behavioral sciences [11, 22, 30, 43]. In the field of health research, in addition to drug use behavior [34, 35], alcohol consumption [9, 35], and sexual initiation [8], this study indicates that the cusp catastrophe modeling also can be applied to and characterize cigarette smoking among adolescents. Moreover, the success of cusp catastrophe modeling in this study focusing on relationship between cognitive process of PMT and cigarette smoking also imply the potential to apply the same analytical approach to other research of health behaviors related to a number of behavioral theories, such as Social Learning Theory, Problem-Behavior Theory, and theory of triadic influence (TTI). Although successful modeling analysis is showed in this paper and other studies and many behavioral scientists have called for applying the promisingly nonlinear approach to overcome the methodological barrier to interpret a health behavior with both linear and nonlinear process, few behavior research and intervention programs have been built on this method [2, 8, 38]. The findings from our research reinforce once more the significance for utilizing QBC-based cusp catastrophe modeling in examining and characterizing behavior with quantum nature.

There are some limitations to this research. Although we considered school size, diversity of training programs and other key factors to ensure the representativeness of study sample, caution should be considered when the results of this study are generalized to other population or places. Moreover, the dependent variable in this investigation related to smoking behaviors may have led to misreporting of information due to social desirability bias. Participants may have underreported smoking frequency in the past 30 days.

## Conclusions

Despite the limitation, our study contrasted cusp catastrophe, linear, and logistic in describing and characterizing smoking behavior using PMT among adolescents and supported the conclusion that adolescent smoking is a quantum process in nature and cusp catastrophe provides a useful method for analyzing this behavior.
